# Hippocampal transcriptome analysis following maternal separation implicates altered RNA processing in a mouse model of fetal alcohol spectrum disorder

**DOI:** 10.1186/s11689-020-09316-3

**Published:** 2020-05-16

**Authors:** Bonnie L. J. Alberry, Christina A. Castellani, Shiva M. Singh

**Affiliations:** 1 Department of Biology, Western University, 1151 Richmond St, London, Ontario N6A 5B7 Canada; 2 McKusick-Nathans Institute, Department of Genetic Medicine, Johns Hopkins University School of Medicine, 733 North Broadway, Baltimore, MD 21205 USA

**Keywords:** Fetal alcohol spectrum disorder, Prenatal alcohol, Hippocampus, Maternal separation, Gene expression, WGCNA

## Abstract

**Background:**

Fetal alcohol spectrum disorders (FASD) are common, seen in 1–5% of the population in the USA and Canada. Children diagnosed with FASD are not likely to remain with their biological parents, facing early maternal separation and foster placements throughout childhood.

**Methods:**

We model FASD in mice via prenatal alcohol exposure and further induce early life stress through maternal separation. We use RNA-seq followed by clustering of expression profiles through weighted gene co-expression network analysis (WGCNA) to analyze transcriptomic changes that result from the treatments. We use reverse transcription qPCR to validate these changes in the mouse hippocampus.

**Results:**

We report an association between adult hippocampal gene expression and prenatal ethanol exposure followed by postnatal separation stress that is related to behavioral changes. Expression profile clustering using WGCNA identifies a set of transcripts, module 19, associated with anxiety-like behavior (*r* = 0.79, *p* = 0.002) as well as treatment group (*r* = 0.68, *p* = 0.015). Genes in this module are overrepresented by genes involved in transcriptional regulation and other pathways related to neurodevelopment. Interestingly, one member of this module, *Polr2a*, polymerase (RNA) II (DNA directed) polypeptide A, is downregulated by the combination of prenatal ethanol and postnatal stress in an RNA-Seq experiment and qPCR validation (*q* = 2e−12, *p* = 0.004, respectively).

**Conclusions:**

Together, transcriptional control in the hippocampus is implicated as a potential underlying mechanism leading to anxiety-like behavior via environmental insults. Further research is required to elucidate the mechanism involved and use this insight towards early diagnosis and amelioration strategies involving children born with FASD.

## Background

Ethanol is a teratogen that crosses the placenta and the blood-brain barrier, disrupting development. Alcohol use during gestation has been associated with various undesirable outcomes, including stillbirth [1], spontaneous abortion [2], premature birth [3], and growth retardation [4]. Fetal alcohol spectrum disorder (FASD) is a direct result of gestational alcohol use, characterized by prenatal and postnatal growth restrictions, facial abnormalities, structural brain abnormalities, microcephaly, developmental delays, intellectual impairment, and behavioral difficulties [5]. Despite societal efforts to raise awareness of these risks, gestational alcohol use persists in North America. In Canada, an estimated 10% of pregnant women consume alcohol [6], and the prevalence of FASD in Canadian 7–9-year-olds is estimated between 2–3% [7]. Similarly, in a cross-sectional study of four communities in the USA, the estimated prevalence of FASD ranged from 1.1 to 5% [8]. The annual cost of FASD in Canada is estimated at approximately $1.8 billion, including costs due to productivity losses, the correctional system, and health care [9]. While preventable, FASD remains a common and costly societal burden throughout an affected individual’s lifetime.

Children with FASD represent a significant proportion of children entering childcare systems such as foster care or orphanages [10]. Compared to a global estimate, the prevalence of FASD for children in care systems is 5.2–67.7 times higher in Canada [11]. An unstable home environment results in a variety of postnatal stresses, often involving stress caused by maternal separation. In fact, exposure to early life stress via neglect or abuse increases the risk of psychiatric disorders later in life [12]. In rodents, early life stress also has a notable effect on hippocampus-specific learning and memory processes [13–15]. Stress activates hippocampal neurons, ultimately leading to increased glucocorticoid receptor signaling [16]. The hippocampus is critical for spatial learning and memory; through synaptic plasticity, it is susceptible to the environment in ways that are often adaptive, although also make it vulnerable to chronic stress. As it stands, little is known about the combination of prenatal alcohol exposure and early life stresses experienced by children with FASD [17]. Children exposed to alcohol in utero and maltreatment during development are more likely to have impaired speech [18], memory, attention, intelligence, and other behavioral deficits [19–21]. The molecular mechanisms involved in this interaction, however, have not been investigated.

Performing detailed molecular research on these topics in humans is challenging, as such we utilize animal models. Given that children with prenatal alcohol exposure often face postnatal chronic stress, we developed an animal model of FASD using C57BL/6J (B6) mice [22]. Further, we have used this model to explore how postnatal stresses associated with maternal separation may compound behavioral and developmental deficits in mice following prenatal alcohol exposure [23]. The results follow the literature and show that pups prenatally exposed to ethanol develop learning deficits, anxiety-like behaviors, and changes in activity [22, 24–26]. Specifically, in the Barnes maze test for learning and memory, following prenatal alcohol exposure and postnatal separation stress, mice were slower to reach the location of a learned target [23]. In the open-field test (OFT), mice are placed in a novel environment to freely explore and exploration of the center zone is indicative of anxiety-like behavior. In this test, mice prenatally exposed to alcohol are quicker to enter the center zone than controls. Finally, the home cage activity test (HC) is used to assess activity in a familiar environment. In the HC test, mice that had faced postnatal maternal separation were less active than controls [23]. Also, early life stress introduced by maternal separation and isolation during early development in mice may lead to increased anxiety-like behaviors in adults [27]. The results of rodent models of maternal separation have found the first 10 postnatal days represent the most critical period [28], as such, separation models have focused on this time [29, 30].

Neurodevelopment lasts into adulthood and can be affected by ethanol exposures and external stresses at any time during this period, suggesting that early postnatal environment can alter adult behavior in progeny that had faced prenatal ethanol exposure [31]. This influence may involve changes in gene expression as the foundation for alterations in neurodevelopment and brain function. Here, we use our mouse model to identify changes in hippocampal gene expression following prenatal ethanol exposure and postnatal maternal separation stress in mice.

## Methods

### Animals

C57BL/6J (B6) mice (*mus musculus*) were obtained from Jackson Laboratories (Bar Harbor, ME, USA) and bred in the Animal Care Facility at Western University (London, Ontario, Canada). Mice were housed in same-sex colonies of up to four individuals with unrestricted access to food and water. Cage, bedding, and nest material were consistent between cages. The colony room operated on a 14:10-h light-dark cycle, with a humidity range from 40 to 60% and a temperature range from 21 to 24 °C.

### Continuous preference drinking model

Following the continuous preference drinking (CPD) model, 10-week-old females were individually housed and randomly assigned to either the control group, dams with free access to water only, or the ethanol group, voluntary ethanol consumption dams with free access to water and a 10% ethanol in water solution [22, 23]. The ethanol group was presented ethanol solution in water with increasing concentrations available from 2%, 5%, and 10%, each introduced after 48 h of exposure to the previous concentration. Following 10% ethanol availability over 10 days, females were mated with 15-week-old males, with only water available. Males were removed after 24 h, representing gestational day 0. Ethanol was available to ethanol females until postnatal day 10. Ethanol was then removed, and only water was provided to all females for the duration of the study. While blood alcohol levels were not taken to minimize maternal stress, voluntary consumption of 10% ethanol at 14 g ethanol per kilogram body weight per day has been shown to produce peak blood alcohol levels of 120 mg dl^−1^ [24]. Experimental mice consumed an average of 8 g ethanol per kilogram body weight per day, representing a modest level of ethanol exposure. This level of exposure has produced modest behavioral deficits in these offspring, including hyperactivity in a novel environment, hypoactivity in a home cage environment, and learning deficits [23].

### Early life stress via maternal separation and isolation

Early life stress via postnatal maternal separation occurred as previously described [23, 32, 33]. Half of the pups in each litter (2–4 pups per litter) were randomly selected for separation stress on postnatal day 2. Tail coloring with permanent marker was used to distinguish pups. Pups were removed and isolated for 3 h per day during the light phase from 10:00 to 13:00 up to and including postnatal day 14 in 8 oz. Dixie cups with bedding and nest material. Pups not selected for separation remained with the dam and other littermates during this time. Weaning occurred at postnatal day 21, with same-sex littermates housed in cages of 2–4 individuals. Following treatments, experimental mice belonged to one of four groups: control, ethanol, stress, or ethanol + stress.

### Hippocampal dissection and RNA isolation

On postnatal day 70, male mice were sacrificed via carbon dioxide asphyxiation and cervical dislocation. The hippocampus was dissected from the whole brain as previously described [34]. Hippocampus samples were transferred to individual tubes containing phosphate-buffered saline (PBS), snap-frozen in liquid nitrogen, and stored at − 80 °C. Using a pestle, samples were ground over liquid nitrogen to create a powder. While on ice, stages of buffer RLT (Qiagen, Valencia, CA) were added and mixed by pipetting. Following 10 min incubation, samples were centrifuged. The supernatant was loaded onto AllPrep DNA spin columns, and the AllPrep DNA/RNA Mini Kit Protocol (Qiagen, Valencia, CA) was followed to isolate DNA and RNA from the same tissue sample. Total RNA was suspended in 100 μL of RNase-free water. RNA quantification was determined by NanoDrop 2000c Spectrophotometer (Thermo Fisher Scientific, Wilmington, DE).

### RNA-Seq

RNA samples were sent on dry ice to The Centre for Applied Genomics (TCAG) (The Hospital for Sick Children, Toronto, Ontario, Canada). The RNA quality was analyzed by Agilent Bioanalyzer 2100 RNA Nano (Agilent Technologies, Santa Clara, CA) by assessing 28S/18S ratios of ribosomal RNA bands. Samples used all had RNA integrity numbers (RINs) greater than 8, indicating they were not degraded. RNA Library preparation followed the Illumina TruSeq Stranded Total RNA Library Preparation protocol to include poly(A) messenger RNA (mRNA) and (lncRNA) using 400 ng total RNA as starting material, with ribosomal RNA (rRNA) depletion using RiboZero Gold. Following RNA fragmenting at 94 °C for 4 min, it was converted to double-stranded complementary DNA (cDNA), end-repaired, and 3′ adenylated for ligation of TruSeq adapters. Sample fragments were amplified with different barcode adapters for multiplex sequencing under the following PCR conditions: 10 s denaturation at 98 °C, 13 cycles of 10 s at 98 °C, 30 s at 60 °C, 30 s at 72 °C, and final extension of 5 min at 72 °C. To confirm fragment size, 1 μL of final RNA library was loaded on a Bioanalyzer 2100 DNA High Sensitivity chip (Agilent Technologies, Santa Clara, CA). Kapa Library Quantification Illumina/ABI Prism Kit protocol (KAPA Biosystems) was used to quantify RNA libraries by qPCR. After pooling in equimolar amounts, libraries were paired-end sequenced on an Illumina HiSeq 2500 platform using a High Throughput Run Mode flowcell and V4 sequencing chemistry following recommended Illumina protocol to generate 126-bp long paired-end reads. Sequence data is available at the Gene Expression Omnibus (GEO), accession number GSE133369. Read quality was assessed using FastQC, with all reads passing the per-base sequence quality and per sequence quality scores analysis modules, indicating minimal degradation and an error rate of less than 0.2%, respectively. Similarly, all reads passed the per base N content and sequence length distribution analysis modules, indicating consistency in quality and sequence length, as expected. The per base sequence content analysis module results for each read have non-uniform base composition, and the sequence duplication levels are higher than FastQC predicts, consistent with RNA libraries. There are overrepresented sequences in each read, although none represents hits in the FastQC database of common contaminants, indicating these are naturally present sequences. None of the reads fail the adapter content analysis module, indicating that no sequence is present in more than 10% of all reads. Finally, as typical with RNA-Seq libraries, there are some highly represented kmers in each read, likely derived from highly expressed sequences.

### Pseudoalignment and differential expression analysis

Paired-end reads for each sample were quantified via pseudoalignment to version 38 of the Ensembl annotation of the mouse transcriptome using kallisto [35]. To estimate the inferential variance of transcript abundance, 100 bootstrap samples were taken. Differential analysis of gene expression was determined using sleuth [36] via transcript *p* value aggregation [37]. The kallisto-sleuth pipeline for differential expression of transcripts via *p* value aggregation was chosen over other methods for its improved sensitivity and conservative false discovery rate [36]. For comparison, HISAT2 [38] and featureCounts [39] were also used to align reads and assign features to version 38 of the Ensembl annotation of the mouse transcriptome. The sleuth object model was defined based on the treatment group, with Wald tests performed to compare each experimental treatment group (ethanol, stress, and ethanol + stress) to the control group. Similarly for comparison, DESeq2 was used for differential analysis of gene expression [40] using aligned reads from the HISAT2-featureCounts pipeline, also defining the model based on the treatment group as when using sleuth. Generalized hypergeometric tests for enrichment of gene ontology (GO) terms and Kyoto encyclopedia of genes and genomes (KEGG) pathways were used for genes represented by transcripts differentially expressed in each treatment group following the kallisto>sleuth pipeline using the goana and kegga functions in the *limma* software package [41] in R, filtered by significance (*p* < 0.05). The background gene set used was created using biomaRt [42] in R to obtain gene IDs from the “mmusculus_gene_ensembl” dataset (mm10) filtered for transcript variants detected in any sample from the RNA-Seq experiment.

### Weighted gene co-expression network analysis

Transcript abundance in transcripts per million (tpm) was used for weighted gene co-expression network analysis (WGCNA) for transcripts detected in all samples (92,187) [43]. As less than 20 samples were used, the soft power threshold was set at 9 to produce adjacency matrices from correlation values for each combination of transcripts. A topological overlap matrix and topological overlap dissimilarity matrix were produced and used for agglomerative hierarchical clustering by the average linkage method. Transcripts were clustered based on the topological overlap between them. Modules were defined using blockwise network analysis with a maximum block size of 15,000, minimum module size of 30, merge cut height of 0.35, with deepSplit at the default 2 for medium sensitivity. Transcripts that did not cluster in a specific module were placed in module 0 and not considered for further analysis. Modules were numerically labeled by module size, with module 1 being the largest module. Each module was represented by the module eigengene (ME), the first principle component of the module.

Co-expression modules were related to 11 traits based on treatment as well as phenotypic results as previously described [23]. Briefly, this includes prenatal ethanol treatment, postnatal stress treatment, experimental group, Barnes maze learning score, weight at postnatal day 21, activity, distance travelled, latency to enter the center zone, and the number of entries into the center zone of the open field test, as well as activity, and the number of rears in the home cage activity test. Each of these traits was experimentally assigned as a treatment or a measured outcome significantly different following at least one of the treatments. Module-trait correlations were filtered by significance (*p* < 0.05). Modules of interest were selected as significantly correlated modules to each trait, resulting in 20 modules of interest. Generalized hypergeometric tests for enrichment of GO terms were used for genes represented by transcripts present in each module using Enrichr [44], filtered by significance (*p* < 0.05). Similarly, KEGG pathways were determined using the kegga function in the *limma* software package [41] in R, filtered by significance (*p* < 0.05).

### qPCR for gene expression

Purified hippocampal RNA was converted to cDNA using the SuperScript IV VILO Master Mix with ezDNase Enzyme following manufacturer’s instructions (Thermo Fisher Scientific). TaqMan Assays were used to investigate the gene of interest, *Polr2a* (ID Mm00839502_m1, FAM labeled), in a multiplex reaction with TATA box binding protein (*Tbp*) as an endogenous reference gene (ID Mm01277042_m1, VIC labeled) with the TaqMan Fast Advanced Master Mix according to the manufacturer’s instructions (Applied Biosystems). The 2^−ΔΔCt^ method was used to assess relative quantity.

## Results

**Fig. 1:**
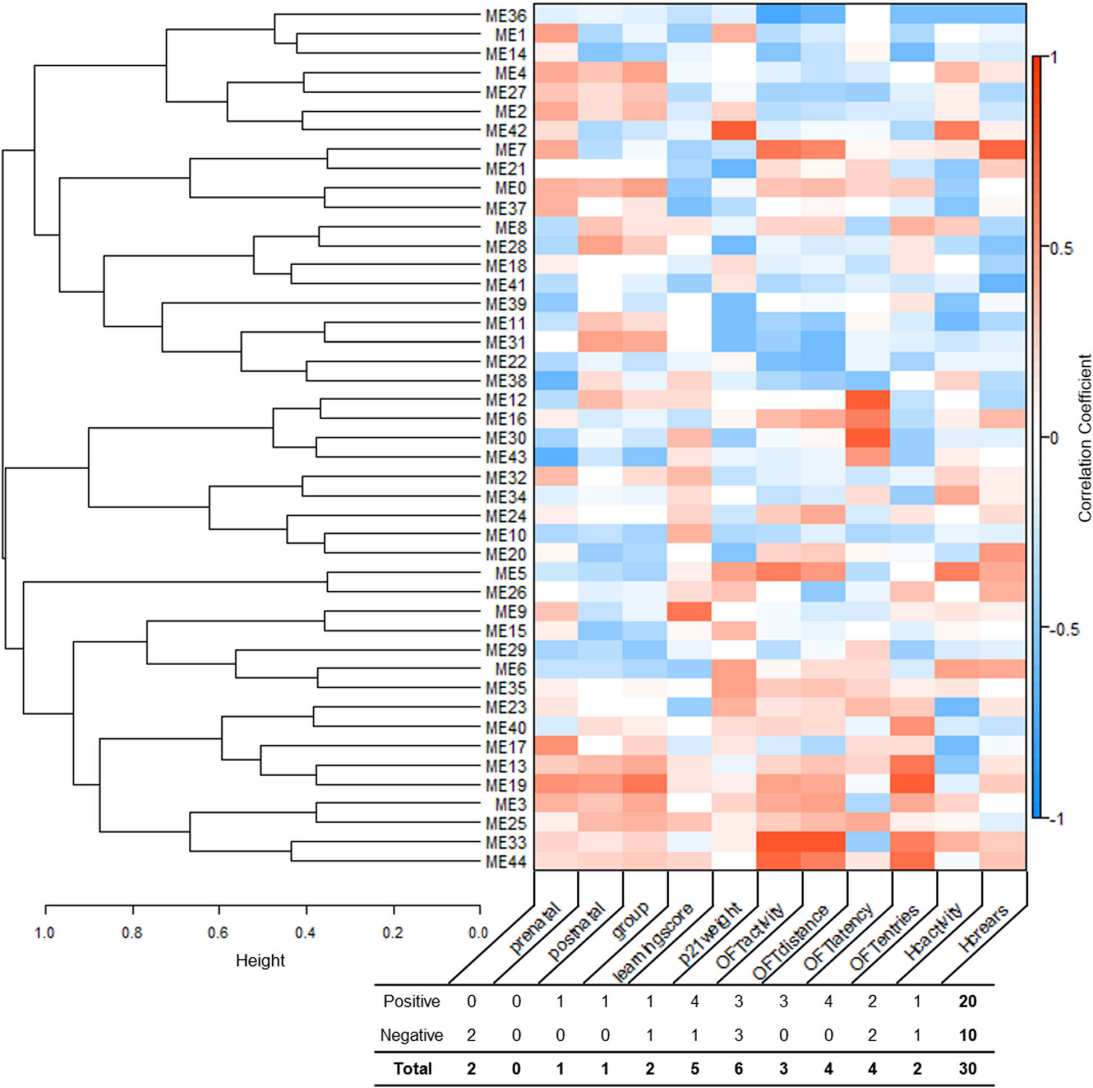
Module formation and trait association using weighted gene co-expression network analysis (WGCNA). Hierarchical clustering dendrogram of module eigengenes with the dissimilarity of eigengenes given by 1-(eigengene correlation) and module-trait correlation heatmap for 11 traits: prenatal or postnatal treatment, experimental group (group), Barnes maze learning score (learningscore), weight at postnatal day 21 (p21weight), open field test activity (OFTactivity), distance (OFTdistance), latency to enter the center (OFTlatency), number of center entries (OFTentries), home cage activity (HCactivity), and number of rears (HCrears), with positive (red) and negative (blue) correlations, with the number of significantly (*p* < 0.05) positively or negatively correlated modules with each trait indicated

RNA-Seq was performed on hippocampal RNA samples from three individuals for each of four groups of mice representing a control group with no experimental interventions, an ethanol group of mice prenatally exposed to ethanol, a stress group with mice subjected to postnatal maternal separation stress, and an ethanol + stress group with mice prenatally exposed to ethanol followed by postnatal maternal separation stress. Transcriptomes from these 12 samples were assessed via RNA-Seq to determine how they differ between treatment groups and control. Reads were pseudoaligned via kallisto and compared to alignment by HISAT2 and featureCounts for feature assignment, with kallisto resulting in between 3.09% fewer to 0.91% more successfully assigned reads of the total number of reads processed for each sample than HISAT2-featureCounts (Supplementary Table 1). Additionally, sample hierarchical clustering indicates no obvious outliers (Supplementary Figure 1). WGCNA was used to cluster transcripts into modules based on correlated expression across samples that can be assessed in relation to other known traits. For optimal correlations between modules and traits of interest, we chose a cutHeight of 0.35. A lower cutHeight results in more modules with fewer genes per module, with adjacent modules sharing trait associations, while increasing cutHeight results in fewer, larger modules. Here, it results in 44 modules, represented by 43 to 11,946 transcripts in each module (Fig. 1).

### WGCNA reveals associations between gene modules and treatment outcomes

We assessed the correlation between each module produced using WGCNA and 11 traits specified either by assignment or previous measurement, including prenatal treatment, postnatal treatment, treatment group, Barnes maze learning score (learningscore), weight on postnatal day 21 (p21weight), open field test measures of activity (OFTactivity), distance (OFTdistance), latency to enter the center (OFTlatency), and the number of entries to the center (OFTentries), as well as home cage measures of activity (HCactivity) and the number of rears (HCrears) [23] (Supplementary Table 2). These traits have unique correlations with modules with visibly shared patterns (Fig. 1). Although some modules share similar correlations, no two traits have the same correlation status with each module. Modules clustering next to one another share similar patterns of correlation between some traits, but no two modules have the same profile at this level. Using a nominally significant cutoff (*p* < 0.05), 30 module-trait relationships emerge, with a range from zero to six significantly correlated modules per trait. Further, most of these relationships (20/30) are positive correlations, while others (10/30) are negative. Also, 20 of the 44 modules (45.56%) are significantly correlated to at least one trait, four correlated to two traits, and three modules correlated to three traits. It is worth noting, however, that these traits are not independent. While there are several overlapping correlations between traits, only one module, module 19 (ME19), is correlated with both experimental treatment and measured behavioral outcome.

### Module 19: RNA polymerase II-associated functions are correlated to the experimental group and anxiety-like behavior

One module, ME19, is correlated with experimental group (group) (*r* = 0.68, *p* = 0.015), as well as number of entries into the center during the open field test (OFTentries) (*r* = 0.79, *p* = 0.002). This module is composed of 895 transcripts that align to 739 annotated genes, including two complete protein complexes (Supplementary Table 3). Epidermal growth factor and its receptor (EGF to EGFR) are represented in this module by *Egf* and *Egfr* transcripts. In addition, the *N*-methyl-d-aspartate (NMDA) glutamate receptor (NMDAR) is represented by *Grin2b*, glutamate receptor, ionotropic, NMDA2B (epsilon 2), and *Grin1*, glutamate receptor, ionotropic, NMDA1 (zeta 1). Expression patterns of module 19 gene transcripts are not driven by individual samples, as indicated by heatmap and hierarchical clustering (Supplementary Figure 2). Using a modest threshold for inclusion (*p* < 0.05), Module 19 genes implicate KEGG pathways important in transcription such as RNA degradation (*p* = 0.006), as well as neurodevelopment and neurodegeneration, including adherens junction (*p* = 0.015) (Table 1, Supplementary Table 3). Major gene ontologies implicated by genes in this module using the same threshold are important for neurodevelopment and neurodegeneration, such as beta-catenin-TCF complex (*p* = 6 × 10^−4^), Notch signaling (*p* = 7 × 10^−4^), and MAPK cascade (*p* = 9 × 10^−4^), as well as transcription, including RNA polymerase II functions (*p* = 0.001) (Table 1, Supplementary Table 3).

**Table 1:** Top 5 most significantly over-represented KEGG pathways and gene ontology (GO) terms represented by genes in module 19

Term	***p*** value
**KEGG pathway**	
RNA degradation	0.0051
Rap1 signaling	0.0079
Prostate cancer	0.0088
Arginine and proline metabolism	0.0102
Adherens junction	0.0111
**Cellular components**	
Beta-catenin-TCF complex	0.0006
Melanosome	0.0009
Pigment granule	0.0009
Azurophil granule lumen	0.0047
Microbody	0.0055
**Biological processes**	
Positive regulation of binding	0.0006
Notch signaling pathway	0.0007
MAPK cascade	0.0009
Regulation of protein targeting to mitochondrion	0.0011
Regulation of protein metabolic process	0.0012
**Molecular functions**	
RNA polymerase II regulatory region sequence-specific DNA binding	0.0010
Chromo shadow domain binding	0.0014
RNA polymerase II distal enhancer sequence-specific DNA binding	0.0025
Ubiquitin-like protein ligase binding	0.0046
RNA polymerase II core promoter proximal region sequence-specific DNA binding	0.0057

### Prenatal ethanol exposure and early life maternal separation stress are associated with changes in gene expression

**Fig. 2:**
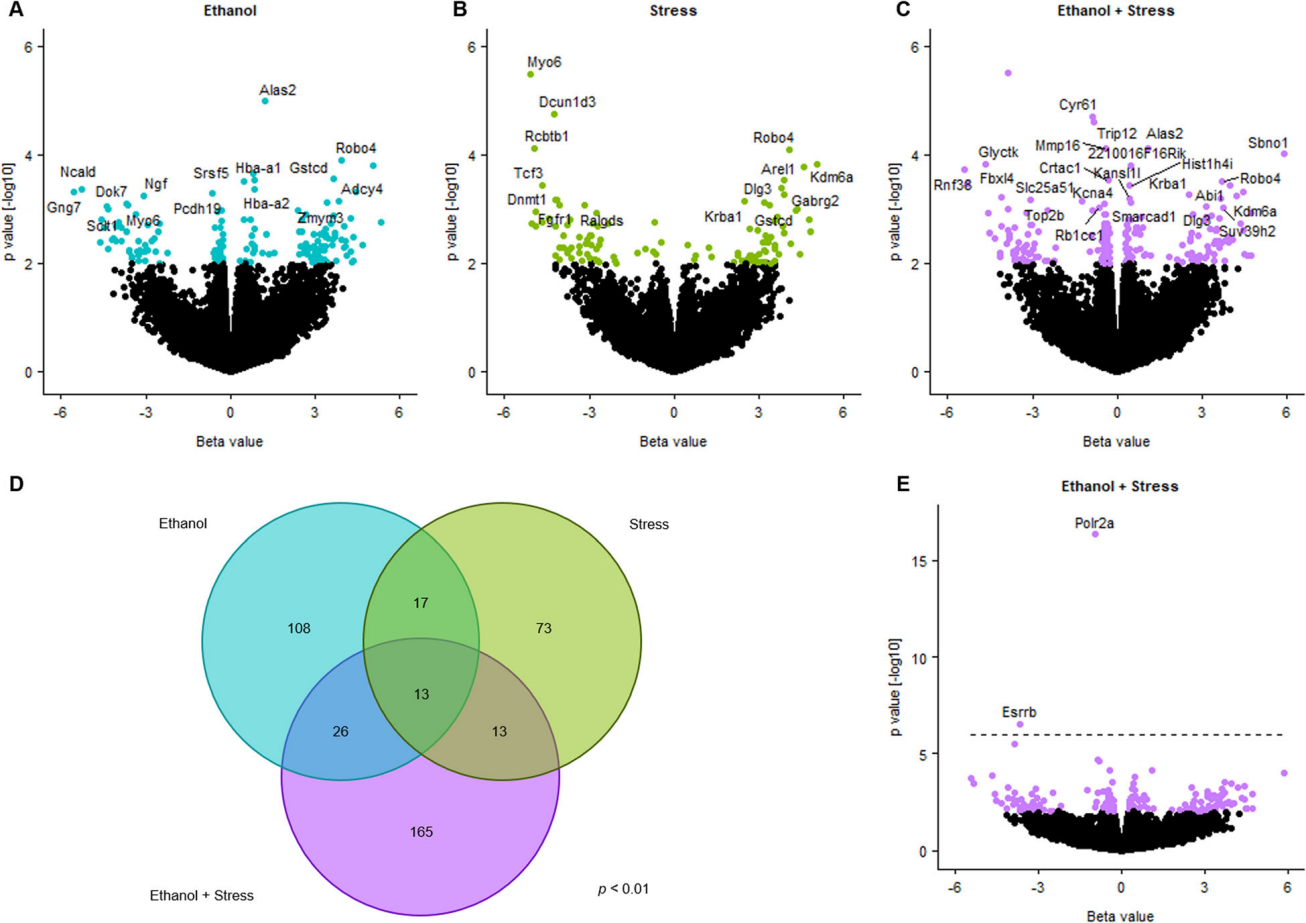
Differential gene expression between groups as detected by sleuth. Volcano plots indicating effect size (beta value) and significance (*p* < 0.01 by color) for each transcript in **a** ethanol, **b** stress, and **c** ethanol + stress groups compared to control treatment with nominally significant (*p* < 0.001), annotated genes labeled. **d** Venn diagram of overlapping transcripts differentially expressed in each treatment as compared to controls (*p* < 0.01). **e** Volcano plot indicating effect size (beta value) and significance for transcripts with an expanded scale and significant (*q* < 0.05) annotated genes labeled for ethanol + stress compared to control, where dashed line indicates the axis cutoff of 6 presented in **c**

Differentially expressed genes were determined at the transcript level for each experimental treatment group compared to the control group via sleuth (Supplementary Table 4). Genes with larger effect size (beta values) tend to also reach greater significance (Fig. 2a–c). Filtering for existing transcripts aligned to the mouse genome (mm10), 164 unique transcripts were implicated by ethanol, 116 by stress, and 217 by the combination of two treatments (*p* < 0.01) (Fig. 2d). There was some overlap between lists, with 13 transcripts shared by all three lists. Differentially expressed genes for each treatment group were analyzed for enrichment of gene ontology and KEGG pathways using annotated genes from these transcripts (Table 2, Supplementary Table 4). Transcripts differentially expressed following prenatal ethanol treatment are important for mRNA processing (*p* < 1.01 × 10^−5^) and synapse localization (*p* < 0.001). Following postnatal maternal separation stress, transcripts important for cell polarity (*p* = 8.64 × 10^−5^) and several neurological pathways (*p* < 0.001) are differentially expressed. For mice exposed to both prenatal ethanol and postnatal stress, altered transcripts are important for stimulus response (*p* < 2.39 × 10^−4^). Taken together, some ontologies and pathways altered by either prenatal ethanol exposure or postnatal stress are shared, particularly with synaptic functions (synapse, synapse part, postsynapse, GABAergic synapse). In addition, genes related to hemoglobin binding have altered expression in the ethanol as well as ethanol + stress groups (*p* < 1.69 × 10^−5^).

**Table 2:** Top 5 most significantly over-represented GO terms and KEGG pathways represented by annotated genes of transcripts differentially expressed in the ethanol, stress, or ethanol + stress groups compared to control (*p* < 0.01)

Ethanol		Stress		Ethanol + stress	
Term	***p*** value	Term	***p*** value	Term	***p*** value
**Molecular function**					
Haptoglobin binding	1.64E−09	Protein binding	8.30E−05	Haptoglobin binding	6.68E−09
Oxygen binding	5.48E−07	Binding	1.28E−04	Oxygen binding	2.17E−06
Hemoglobin alpha binding	1.97E−06	Protein serine/threonine/tyrosine kinase activity	0.001	Hemoglobin alpha binding	4.56E−06
Hemoglobin binding	1.69E−05	Transferase activity	0.002	Hemoglobin binding	3.89E−05
Protein binding	1.84E−05	Catalytic activity, acting on a protein	0.002	Oxygen carrier activity	9.20E−05
**Biological process**					
Regulation of alternative mRNA splicing, via spliceosome	3.75E−07	Microtubule cytoskeleton organization involved in establishment of planar polarity	8.64E−05	Macromolecule modification	2.34E−04
Regulation of mRNA splicing, via spliceosome	8.29E−07	Golgi organization	4.85E−04	Response to chemical	2.37E−04
Alternative mRNA splicing, via spliceosome	1.25E−06	Endomembrane system organization	0.001	Response to temperature stimulus	2.39E−04
Regulation of RNA splicing	8.49E−06	Embryo development	0.001	Cellular protein modification process	2.55E−04
Regulation of mRNA processing	1.01E−05	Organonitrogen compound metabolic process	0.001	Protein modification process	2.55E−04
**Cellular component**					
Hemoglobin complex	1.64E−09	Intracellular part	7.05E−05	Hemoglobin complex	6.68E−09
Haptoglobin-hemoglobin complex	3.67E−09	Intracellular	9.42E−05	Haptoglobin-hemoglobin complex	1.49E−08
Synapse	2.09E−05	Organelle part	1.43E−04	Intracellular part	1.39E−04
Synapse part	2.86E−05	Intracellular organelle part	3.46E−04	Intracellular	2.07E−04
Postsynapse	0.001	Basal cortex	0.001	Cell part	0.001
**KEGG pathway**					
African trypanosomiasis	6.90E−06	Circadian entrainment	0.002	African trypanosomiasis	2.66E−05
Malaria	3.50E−05	Retrograde endocannabinoid signaling	0.009	Malaria	1.31E−04
Steroid biosynthesis	0.01	Glycerophospholipid metabolism	0.012	Mucin type O-glycan biosynthesis	0.002
Porphyrin and chlorophyll metabolism	0.028	GABAergic synapse	0.012	Glycine, serine, and threonine metabolism	0.008
ECM-receptor interaction	0.029	Morphine addiction	0.012	Systemic lupus erythematosus	0.008

Some overlap exists between these three lists, specifically when examining the top 25 most significant annotated transcripts in each list (Table 3). Most notably, *Robo4*, Roundabout guidance receptor 4, and *Krba1*, KRAB-A domain containing 1, are upregulated in each treatment group, with effect sizes ranging from 2.40 to 4.07. *Alas2*, aminolevulinic acid synthase 2, erythroid, is the most significantly differentially expressed gene following ethanol treatment (beta = 1.21, *p* = 1.04 × 10^−5^) and is also upregulated in the ethanol + stress group (beta = 1.08, *p* = 7.71 × 10^−5^). Similarly, *Suv39h2*, suppressor of variegation 3-9 2, is also upregulated following prenatal ethanol exposure (beta = 3.64, *p* = 1.34 × 10^−3^) as well as following the combined ethanol + stress treatments (beta = 3.74, *p* = 9.76 × 10^−4^). *Kdm6a*, lysine (K)-specific demethylase 6a, *Sbno1*, strawberry notch 1, and *Dlg3*, discs large MAGUK scaffold protein 3, are shared between the stress and ethanol + stress groups as upregulated when compared to controls.

**Table 3:** Top 25 annotated gene transcripts identified in each treatment group, where beta value represents the effect size for each transcript detected

Ethanol			Stress			Ethanol + stress		
Gene	***p*** value	Beta	Gene	***p*** value	Beta	Gene	***p*** value	Beta
*Alas2*	1.04E−05	1.21	*Myo6*	3.34E−06	− 5.06	*Polr2a*	4.71E−17	− 0.96
*Robo4*	1.27E−04	3.95	*Dcun1d3*	1.80E−05	− 4.24	*Esrrb*	2.88E−07	− 3.66
*Gstcd*	2.83E−04	3.66	*Rcbtb1*	7.47E−05	− 4.94	*Cyr61*	2.03E−05	− 0.86
*Hba-a1*	2.91E−04	0.86	*Robo4*	8.00E−05	4.07	*Trip12*	2.56E−05	− 0.82
*Srsf5*	3.17E−04	0.46	*Kdm6a*	1.66E−04	4.59	*Alas2*	7.71E−05	1.08
*Hba-a2*	4.24E−04	0.84	*Tcf3*	3.60E−04	− 4.65	*Mmp16*	7.72E−05	− 0.41
*Ncald*	4.43E−04	− 5.29	*Arel1*	4.23E−04	3.77	*Sbno1*	9.86E−05	5.88
*Gng7*	4.83E−04	− 5.57	*Gabrg2*	5.55E−04	3.89	*Glyctk*	1.47E−04	− 4.66
*Pcdh19*	5.28E−04	− 0.66	*Dnmt1*	6.76E−04	− 4.19	*2210016F16Rik*	1.56E−04	0.49
*Ngf*	5.62E−04	− 3.09	*Fgfr1*	6.79E−04	− 4.13	*Rnf38*	1.90E−04	− 5.42
*Adcy4*	7.04E−04	3.82	*Krba1*	7.19E−04	2.49	*Crtac1*	2.97E−04	− 0.33
*Zmym3*	7.77E−04	3.40	*Dlg3*	7.68E−04	3.16	*Robo4*	3.13E−04	3.72
*Dok7*	7.88E−04	− 3.70	*Ralgds*	8.60E−04	− 3.16	*Abi1*	3.72E−04	3.96
*Myo6*	8.78E−04	− 3.62	*Gstcd*	8.61E−04	3.36	*Hist1h4i*	3.76E−04	0.45
*Sclt1*	8.91E−04	− 4.40	*Stx5a*	1.05E−03	4.30	*Krba1*	5.56E−04	2.54
*Slitrk2*	1.08E−03	− 0.32	*Tnpo3*	1.15E−03	− 4.89	*Kdm6a*	5.79E−04	4.20
*Krba1*	1.09E−03	2.40	*Haghl*	1.20E−03	− 2.76	*Fbxl4*	6.02E−04	− 4.11
*Arpp21*	1.25E−03	2.66	*Per3*	1.38E−03	3.64	*Kansl1l*	6.41E−04	0.41
*Slc25a40*	1.29E−03	− 3.34	*0610010F05Rik*	1.46E−03	− 4.43	*Top2b*	6.87E−04	− 3.07
*Suv39h2*	1.34E−03	3.64	*Sbno1*	1.58E−03	4.77	*Slc25a51*	7.24E−04	− 1.25
*Bcorl1*	1.50E−03	4.26	*Tmem194b*	1.59E−03	− 3.66	*Smarcad1*	7.47E−04	0.48
*Itgb5*	1.55E−03	− 4.57	*Tpp2*	1.77E−03	− 4.64	*Kcna4*	8.24E−04	− 0.44
*Msi1*	1.55E−03	0.48	*Pnpla6*	1.88E−03	− 5.01	*Dlg3*	8.87E−04	3.12
*A230050P20Rik*	1.61E−03	0.70	*Mettl5*	1.89E−03	− 2.34	*Rb1cc1*	9.35E−04	− 0.64
*Slc17a5*	1.71E−03	− 0.34	*Gtf2a2*	1.90E−03	− 2.87	*Suv39h2*	9.76E−04	3.74

For comparison, following HISAT2 alignment and featureCounts for feature assignment, DESeq2 was used to determine if the top 25 most significantly differentially expressed genes determined by sleuth were replicable using a different analysis pipeline (Supplementary Table 5). For the ethanol group, three genes among the top 25, *Alas2*, *Hba-a1*, hemoglobin alpha, adult chain 1, and *Hba-a2*, hemoglobin alpha, adult chain 2, also have significantly increased expression (*q* < 0.01) in the DESeq2 analysis. For the stress group, none of the top 25 genes determined by sleuth was significantly differentially expressed using DESeq2. For the ethanol + stress group, five of the top 25 genes determined by sleuth, *Alas2*, *Mmp16*, *matrix metallopeptidase 16*, *2210016F16Rik*, RIKEN cDNA 2210016F16 gene, *Crtac1*, cartilage acidic protein 1, and *Kcna4*, potassium voltage-gated channel, shaker-related subfamily, member 4, were also significantly (*q* < 0.01) differentially expressed in the same direction of change using DESeq2. For global differential expression in each treatment comparison using DESeq2, no genes reach significance using a Benjamini-Hochberg corrected *p* value (*q* value) cutoff of 0.05. By employing a more liberal threshold (*p* < 0.01), 59, 12, and 103 genes are differentially expressed as detected by DESeq2 following ethanol, stress, or ethanol + stress treatments, respectively (Supplementary Table 6). Differentially expressed genes (*p* < 0.01) as detected by DESeq2 following each treatment show minimal overlap, with just two genes shared between all three treatments, *Alas2* and *Cpne7*, copine VII (Supplementary Figure 3). All detected genes for each treatment group were *p* value-ranked following each analysis pipeline and compared. There are moderate correlations in gene rank between the kallisto-sleuth pipeline and the HISAT2-featureCounts-DESeq2 pipeline, with significant (*p* < 2.2 × 10^−16^) correlation coefficients of 0.434, 0.428, and 0.573 for ethanol, stress, and ethanol + stress, respectively (Supplementary Figure 3).

**Fig. 3:**
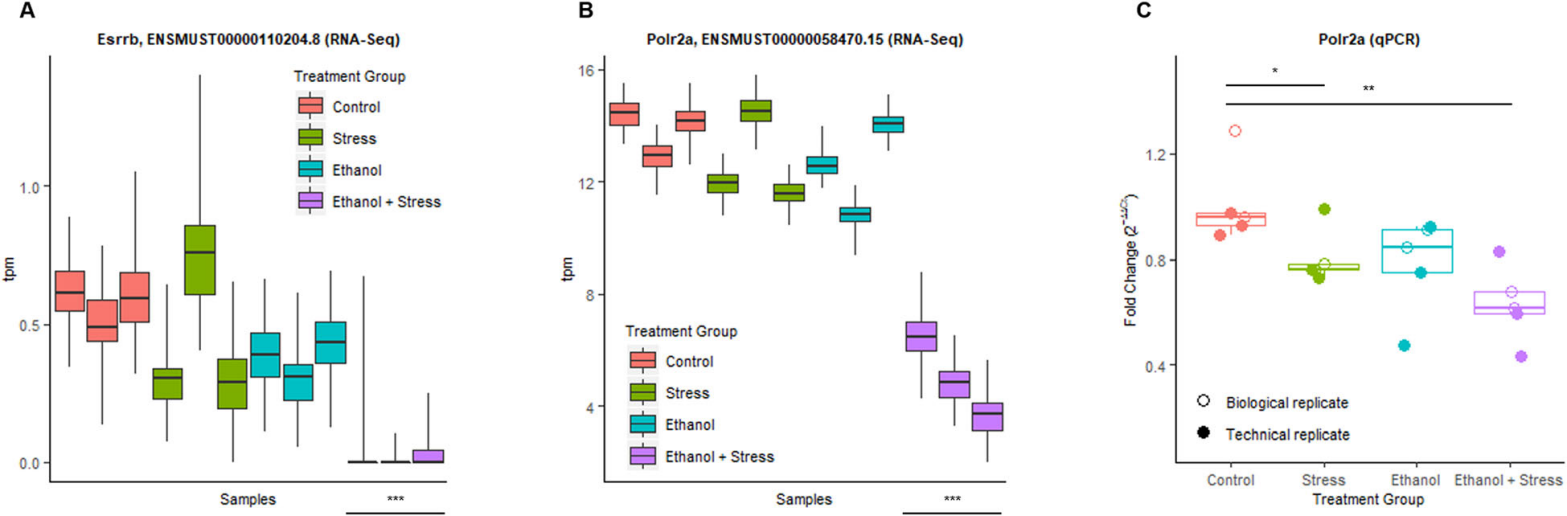
Transcript-specific differential gene expression following ethanol + stress treatment. Transcript abundance in transcripts per million (tpm ± inferential variance) as detected by sleuth from RNA-Seq for **a***Esrrb* and **b***Polr2a*, ***q value < 0.05. **c** The relative quantity of *Polr2a* is decreased 1.24-fold with postnatal stress alone and 1.59-fold when mice were prenatally exposed to ethanol and postnatal stress, as detected by reverse transcription qPCR, *p < 0.05, **p < 0.01. Open circles represent biological replicates that were not used in the RNA-Seq experiment, while closed circles represent samples included in the RNA-Seq experiment and serve as technical replicates

Using a *p* value cutoff adjusted for the false discovery rate (FDR) (*q* < 0.05), ethanol or stress alone does not result in any transcripts with robust changes in differential expression. However, two genes show robust changes in differential expression (*q* < 0.05) between the ethanol + stress group compared to the control group, *Esrrb*, estrogen-related receptor, beta, and *Polr2a*, polymerase (RNA) II (DNA directed) polypeptide A (Fig. 2e). Each of these is the result of a single transcript downregulated following the combination of ethanol + stress (Fig. 3a, b). Interestingly, these two genes are also members of Module 19 from WGCNA. The low level of *Esrrb* expression in the RNA-Seq experiment excluded it from further validation. Downregulation of *Polr2a* was validated by qPCR displaying 1.28-fold decrease in expression following stress (*p* = 0.048) and 1.59-fold decrease following the combination of ethanol + stress (*p* = 0.004) (Fig. 3c).

For *Esrrb*, there are six known expressed transcript variants, five of which are protein-coding. Again, the largest, Esrrb-203 (ENSMUST00000110204.8) (4196 bp) is the transcript described above as differentially expressed between the ethanol + stress group and controls. Other variants are detected in different groups (Supplementary Figure 4), including the expression of Esrrb-206 in samples within each group. Interestingly, Esrrb-204 was only detected in the ethanol + stress group. While there is limited evidence of isoform switching in mice, humans have three forms of ESRRB, likely with a distinct function. The short form is most homologous to mouse ESRRB, and there is evidence that these variants may regulate cell cycle progression differently [45]. Regarding *Polr2a*, in mice, there are four known expressed transcripts, two of which are protein-coding. The larger, Polr2a-201 (ENSMUST00000058470.15) (6740 bp) is the transcript characterized above. In humans, there is only one protein-coding variant homologous to this gene. Interestingly, most of the non-coding isoforms are minimally detected in all groups; however, the other protein-coding variant, Polr2a-202, is expressed in each sample following ethanol + stress treatment (Supplementary Figure 4). Finally, as a comparison measure, the expression patterns of *Polr2a* determined using the DESeq2 pipeline are remarkably similar to the sleuth pipeline using the same RNA-Seq data and qPCR including independent biological samples. Specifically, using the HISAT2-featureCounts-DESeq2 pipeline, *Polr2a* has a fold change of − 0.80 (*p* = 0.041) following ethanol + stress as compared to control (Supplementary Figure 4).

## Discussion

FASD is a complex societal burden that while preventable remains common. Besides prenatal exposure to alcohol, children born with FASD are often also exposed to a stressful postnatal upbringing that invariably includes early maternal separation. The main objective of this study was to understand how early life stresses may complicate molecular changes and behavioral deficits following exposure to prenatal alcohol. The experimental design used has previously assessed the behavioral changes that result from prenatal alcohol exposure and early postnatal stress [23], while the molecular changes reported here are focused on the adult hippocampal transcriptome. The results show that the manifestation of FASD-related deficits may be compounded by additional maternal separation stress via alterations in hippocampal gene expression. Specifically, the analysis of this multifaceted data by WGCNA has led to the identification of modules of genes similarly expressed between samples that correlate with treatment and behavioral outcomes. It has identified one module of genes, module 19, that correlates with treatment and anxiety-like behavior in the progeny (number of center entries in the open field test). Genes in this module are related to transcription and neurodevelopment through various gene ontologies and KEGG pathways. We also performed a transcript-level differential expression analysis, finding that early life stress, as well as the combination of prenatal ethanol exposure and postnatal stress, results in the downregulation of a transcript responsible for an RNA polymerase II subunit (*Polr2a*) in the hippocampus. Together, these results suggest that changes in behavior following prenatal alcohol exposure and postnatal stress likely result from altered hippocampal gene expression.

### WGCNA reveals FASD-relevant module of co-expressed transcripts

We have previously described behavioral changes in these mice as significantly different between treatment groups [23], and here add to that data through the analysis of RNA-Seq gene expression results from the adult hippocampus of the same mice as well as the novel use of WGCNA in an in vivo FASD model. WGCNA is a valuable systems biology tool for the determination of modules of correlated gene expression and relation of these modules to sample traits [43]. WGCNA has identified a single module of hippocampal gene expression, module 19, that is correlated with experimental treatment as well as with a reliably measured phenotypic outcome. In this case, the number of entries into the center zone of the open field test by mice is analogous to an anxiety-like behavior [46, 47]. Interestingly, several FASD-related deficits, including learning, memory, stress response, and anxiety-like behaviors, are under hippocampal control. Specifically, increased adult hippocampal neurogenesis has been associated with reduced anxiety-like behavior in mice [48], and stress-induced alterations in hippocampal microglia have also been associated with anxiety-like behavior [49]. In addition, exposure to chronic stress is associated with activated hippocampal microglia in rats [50]. Such results argue that the WGCNA has identified a set of genes represented in module 19 that are related to the interplay between prenatal ethanol exposure, stress, and anxiety-like behavior. Further, these genes may be relevant to the etiology of FASD.

Next, we focused on the set of genes represented in module 19. We argue that the genes affected represent pathways that may be disturbed in FASD. Genes responsible for the MAPK cascade (21 genes) and notch signaling (8 genes) are included in this module. MAPK signaling has been identified as a strong candidate pathway in FASD and is fundamental to fetal development [51]. MAPK signaling cascade has also been implicated in ethanol-induced apoptosis through the activation of p53 signaling in neural crest cells [52]. In addition, human neural precursors display impaired neurogenesis following alcohol exposure via the downregulation of MAPK genes [53]. The MAPK pathway is also involved in chronic stress, with increased negative regulation via MAPK phosphatase-1 in the hippocampus of chronically stressed rodents [54]. Together, it is unsurprising to find the MAPK cascade implicated by genes represented in module 19, with expression correlated to prenatal alcohol exposure and maternal separation stress as well as anxiety-like behavior in the resulting progeny. Further, genes involved in Notch signaling are also overrepresented in module 19. Notch signaling is important for neurogenesis and embryonic development through its influence on the expression of associated transcription factors, even in the adult brain [55]. Notch signaling has also been altered in prenatal alcohol exposure models involving mice [56] and zebrafish [57]. Our results further support the notion that MAPK and notch signaling, alongside other signaling cascades, implicate a role for altered transcriptional control in the adult hippocampus following prenatal alcohol exposure and early life stress.

### Gene expression patterns implicate neurodevelopmental dysregulation in FASD

To the best of our knowledge, this is the first report of genome-wide changes in hippocampal gene expression following a continuous preference exposure of alcohol in a mouse model of FASD. Our results argue that prenatal ethanol exposure and early maternal separation lead to alterations in specific hippocampal genes depending on the type and combination of exposures. Of interest to this discussion are two transcripts, *Robo4* and *Krba1*, that are among the most significantly altered transcripts in each treatment group, both upregulated in every group compared to control (*p* < 0.001). The protein product for *Robo4* is the roundabout (Robo) receptor, with interacts with the astrocyte-secreted slit guidance ligand 2 (Slit2) during central nervous system development, specifically as an axon repellant during axon guidance [58]. *Krba1* is predicted to regulate transcription via DNA template binding as a zinc-finger protein that represses RNA polymerase promoters [59] and has been associated with gestational long-term exposure to air pollution [60]. If the upregulation of these two genes following each of our treatments results in decreased viability, abnormal synapse formation, or improper transcriptional regulation during neurodevelopment, they may contribute to observed behavioral deficits in FASD. While the function of *Robo4* and *Krba1* in the adult hippocampus is unclear, they are both constitutively expressed in the hippocampus as reported in the Allen Mouse Brain Atlas [61]. The dysregulation of these genes may occur during neurodevelopment and persist into adulthood but may also be relevant in the adult stage.

We also note that two transcripts, *Alas2* and *Suv39h2*, are among the most significantly altered transcripts in the two ethanol treatment groups (ethanol and ethanol + stress) as detected by sleuth. Additionally, *Alas2* is also consistently altered in every treatment using the second independent analytical pipeline, HISAT2-featureCounts-DESeq2. There have been mixed reports regarding environmental exposures and *Alas2* expression, including downregulation following voluntary maternal ethanol consumption [62] or stress exposure in heavy drinkers [63], and upregulation following lead exposure [64] or repeated stress [65]. The protein product of *Suv39h2* is a histone methyltransferase, specifically for H3K9, often resulting in trimethylation leading to inhibition of gene expression [66]. Differential histone methylation in the brain has also been implicated in FASD [67, 68].

Finally, three transcripts, *Kdm6a*, *Sbno1*, and *Dlg3*, were among the most significantly altered transcripts in response to the stress treatments (stress and ethanol + stress groups). *Kdm6a* encodes a histone demethylase that removes suppressive chromatin marks and controls gene expression in microglia for clearance of dying neurons and non-functional synapses [69]. *Sbno1* is a nuclear-localized transcriptional regulator important in Notch and Hippo signaling [70], which has been associated with schizophrenia [71, 72] and may be important for brain function. *Dlg3* encodes synapse-associated protein 102 and is important for synapse formation in the brain, whereby knockout mice have synaptic plasticity impairments, including impaired spatial learning [73], and mutations in DLG3 cause non-syndromic X-linked intellectual disability [74]. We report upregulation of a *Dlg3* transcript in the stress and ethanol + stress groups compared to control, while other research has shown that overexpression inhibits proliferation and induces apoptosis [75]. Taken together, the upregulation of these three genes in the stress and ethanol + stress groups may lead to altered brain transcriptional regulation and synaptic plasticity required for normal learning processes and brain function.

### Gene expression dysregulation compliments WGCNA results

The molecular functions most significantly enriched by Module 19 genes involve RNA polymerase II DNA binding. These are genes correlated with the experimental treatment group and anxiety-like behavior. When looking for differentially expressed genes, we also find that the most significantly differentially expressed transcript coding for an RNA polymerase II subunit (*Polr2a*) is downregulated following stress and ethanol + stress as compared to control. *Polr2a*, polymerase (RNA) II (DNA directed) polypeptide A, encodes for the largest subunit of RNA polymerase II, an enzyme responsible for mRNA synthesis. Like the combination of ethanol + stress, we present here a mouse model of stress + morphine from postnatal days 5 to 9 found decreased *Polr2a* expression following stress alone [76]. Conversely, when combined with morphine treatment, there was an associated upregulation of *Polr2a*, suggesting expression of this gene is particularly sensitive to environmental insults. Ours is not the first evidence of *Polr2a* alterations in FASD research, in a mouse model of binge-like prenatal ethanol exposure, increased promoter DNA methylation of *Polr2a* was found in the hippocampus [67]. Additionally, *Polr2a* expression is decreased in the dentate gyrus of the hippocampus alongside increased anxiety-like behavior following induced glucocorticoid receptor overexpression [77]. WGCNA has also been used to create gene co-expression modules related to ethanol exposure in human embryonic stem cells to help understand molecular mechanisms underlying FASD. Despite the vastly different experimental designs, one module associated with ethanol treatment in the stem cells was also most significantly enriched with RNA polymerase II activity [78].

## Conclusions

We have examined RNA isolated from the adult hippocampus, a more focused tissue type than whole brain homogenate, representing RNA from a mixed cell population. Multiple lines of evidence suggest microglia and neurons respond differently and need to be assessed separately. In our WGCNA analysis, Module 19 was associated with changes in hippocampal gene expression and anxiety-like behavior, an association potentially driven by microglia. Additionally, the *Kdm6a* histone demethylase found differentially expressed in stress and ethanol + stress groups is important for microglia function in the clearance of dying neurons. In fact, developmental alcohol exposure leads to lasting disruption of microglia in rodents [79]. Further work in this area should focus on cell type differences that underlie the observations described here. Also, alterations in gene expression in this study were assessed at postnatal day 70, following prenatal ethanol exposure and/or postnatal stress up to postnatal day 14. It seems unlikely that the direct effect of the treatments used persists 8 weeks later. It is logical to argue that these exposures are causing long-lasting transcriptomic effects. In this context, epigenetic mechanisms are known to be involved in the programming of gene expression throughout development, and as mediators of experience to finely control expression. As in our results, alterations to epigenetic marks such as DNA methylation, histone modifications, and microRNA expression have been implicated following prenatal alcohol exposure and early life stress. As such, future experiments should aim to classify alterations in epigenetic terms that may be responsible for the persistent changes in gene expression seen over a long period. Finally, we have demonstrated that transcriptome changes persist in the hippocampus of adult mice prenatally exposed to alcohol and/or postnatally exposed to early life stress. Some transcripts are co-expressed in a way that correlates with experimental treatment and behavioral outcomes. These transcripts code for genes important in RNA processing and management throughout neurodevelopment and beyond. In addition, some transcripts are significantly differentially expressed between treatment groups. Specifically, the largest protein-coding variant of *Polr2a* (transcript Polr2a-201, ENSMUST00000058470.15) is downregulated following early life stress as well as the combination of prenatal ethanol exposure and postnatal stress. These lasting alterations in transcripts responsible for RNA processing likely underlie the behavioral deficits observed in these mice. They suggest that postnatal stresses further complicate the effects caused by prenatal alcohol exposure in FASD. Further understanding of how these changes occur and persist may result in earlier detection, prognosis, and amelioration in humans faced with FASD.

## Supplementary information

**Additional file 1: Supplementary Table 1.** Read mapping statistics following RNA-Seq and pseudoalignment of reads via kallisto, alignment via HISAT2 and feature association using featureCounts.

**Additional file 2: Supplementary Figure 1. Module creation by weighted gene co-expression network analysis.** (A) Principal component analysis plot for principal components one and two. (B) Sample clustering to detect outliers. (C) Connectivity analysis of the scale-free topology fit for different soft-thresholding powers where numbers indicate the soft-thresholding power (D) mean connectivity of the network for different soft-thresholding powers, a soft-threshold of 9 was used here. (E, F, G, H, I, J, K, L) Transcript similarity clustering dendrograms for blockwise analysis for blocks 1-8, respectively, by clustering of transcripts based on topological overlap with different modules indicated by color below.

**Additional file 3: Supplementary Table 2.** Correlation coefficients and p-values of module-trait associations for each of the 44 modules produced by WGCNA and 11 traits.

**Additional file 4: Supplementary Table 3.** Genes implicated by transcripts in Module 19 associated with the experimental treatment group and the number of center zone entries in the open field test; GO terms and KEGG pathways overrepresented (*p* < 0.05) in module 19.

**Additional file 5: Supplementary Figure 2.** Hierarchical clustering and heatmap of Module 19 gene transcript expression for each sample.

**Additional file 6: Supplementary Table 4.** Differentially expressed gene lists for Ethanol, Stress, and Ethanol + Stress as compared to controls via the kallisto-sleuth pipeline, filtered by significance (*p* < 0.01); GO terms and KEGG pathways overrepresented (*p* < 0.05) by transcripts significantly differentially expressed (*p* < 0.01) for each comparison.

**Additional file 7: Supplementary Table 5.** Top 25 genes for Ethanol, Stress, and Ethanol + Stress as compared to controls via the kallisto-sleuth pipeline found using the HISAT2-featureCounts-DESeq2 pipeline.

**Additional file 8: Supplementary Table 6.** Differentially expressed gene lists for Ethanol, Stress, and Ethanol + Stress as compared to controls via the HISAT2-featureCounts-DESeq2 pipeline, filtered by significance (*p* < 0.01).

**Additional file 9: Supplementary Figure 3.** Venn diagrams of overlapping differentially expressed genes (*p* < 0.01) for (A) each treatment group as detected by DESeq2, (B) Ethanol group as detected by sleuth and DESeq2, (C) Stress group as detected by sleuth and DESeq2, (D) Ethanol + Stress as detected by sleuth and DESeq2. Gene rank by *p*-value density plots between sleuth (x-axis) and DESeq2 (y-axis) analysis pipelines for (E) Ethanol (r = 0.434, *p* < 2.2 x 10^-16^), (F) Stress (r = 0.428, *p* < 2.2 x 10^-16^), and (G) Ethanol + Stress (r = 0.573, *p* < 2.2 x 10^-16^).

**Additional file 10: Supplementary Figure 4.** Transcript abundance in transcripts per million (tpm) as detected by sleuth from RNA-Seq for (A) Esrrb-203 (ENSMUST00000110204.8) and (B) Polr2a-201 (ENSMUST00000058470.15). (C) The relative quantity of *Polr2a* is decreased 1.24 -fold with postnatal stress alone, and 1.59-fold when mice were prenatally exposed to ethanol and postnatal stress, as detected by reverse transcription qPCR, open circles represent biological replicates not used in the RNA-Seq experiment, while closed circles represent samples included in the RNA-Seq experiment and serve as technical replicates. Transcript abundance in transcripts per million (tpm) using the kallisto-sleuth pipeline for all detected transcript variants of (D) *Esrrb* and (E) *Polr2a*. Esrrb-203 (ENSMUST00000110204.8) and Polr2a-201 (ENSMUST00000058470.15) are down-regulated following Ethanol + Stress as compared to controls and represent the largest protein-coding transcripts for each gene. (F) *Polr2a* is decreased 0.80-fold when mice were prenatally exposed to ethanol and postnatal stress, presented as normalized counts determined using the HISAT2-featureCounts-DESeq2 pipeline, *p < 0.05, **p < 0.01, ***q < 0.01. Note: We have included panels A-C from Fig. 3 to facilitate comparison.

## Data Availability

Sequence data is available at GEO, accession number GSE133369.

## Abbreviations

cDNA: Complementary DNA
CPD: Continuous preference drinking model
ECM: Extracellular matrix
EGF: Epidermal growth factor
EGFR: Epidermal growth factor receptor
FASD: Fetal alcohol spectrum disorder
FDR: False discovery rate
GABA: Gamma-aminobutyric acid
GEO: Gene Expression Omnibus
GO: Gene ontology
GWAS: Genome-wide association study
HC: Home cage activity test
KEGG: Kyoto encyclopedia of genes and genomes
lncRNA: Long non-coding RNA
MAPK: Mitogen-activated protein kinase
ME: Module eigengene
mRNA: Messenger RNA
NMDA: *N*-Methyl-d-aspartate
NMDAR: NMDA receptor
OFT: Open field test
qPCR: Real-time (quantitative) polymerase chain reaction
RIN: RNA integrity number
RNA-Seq: RNA sequencing
rRNA: Ribosomal RNA
TCF: T cell factor
TPM: Transcripts per million
WGCNA: Weighted gene co-expression network analysis
