# Supplementary figures and images for: Hippocampal transcriptome analysis following maternal separation implicates altered RNA processing in a mouse model of fetal alcohol spectrum disorder
Source: J Neurodev Disord. 2020 May 16;12:15. doi: 10.1186/s11689-020-09316-3 (PMC7231420; doi:10.1186/s11689-020-09316-3)

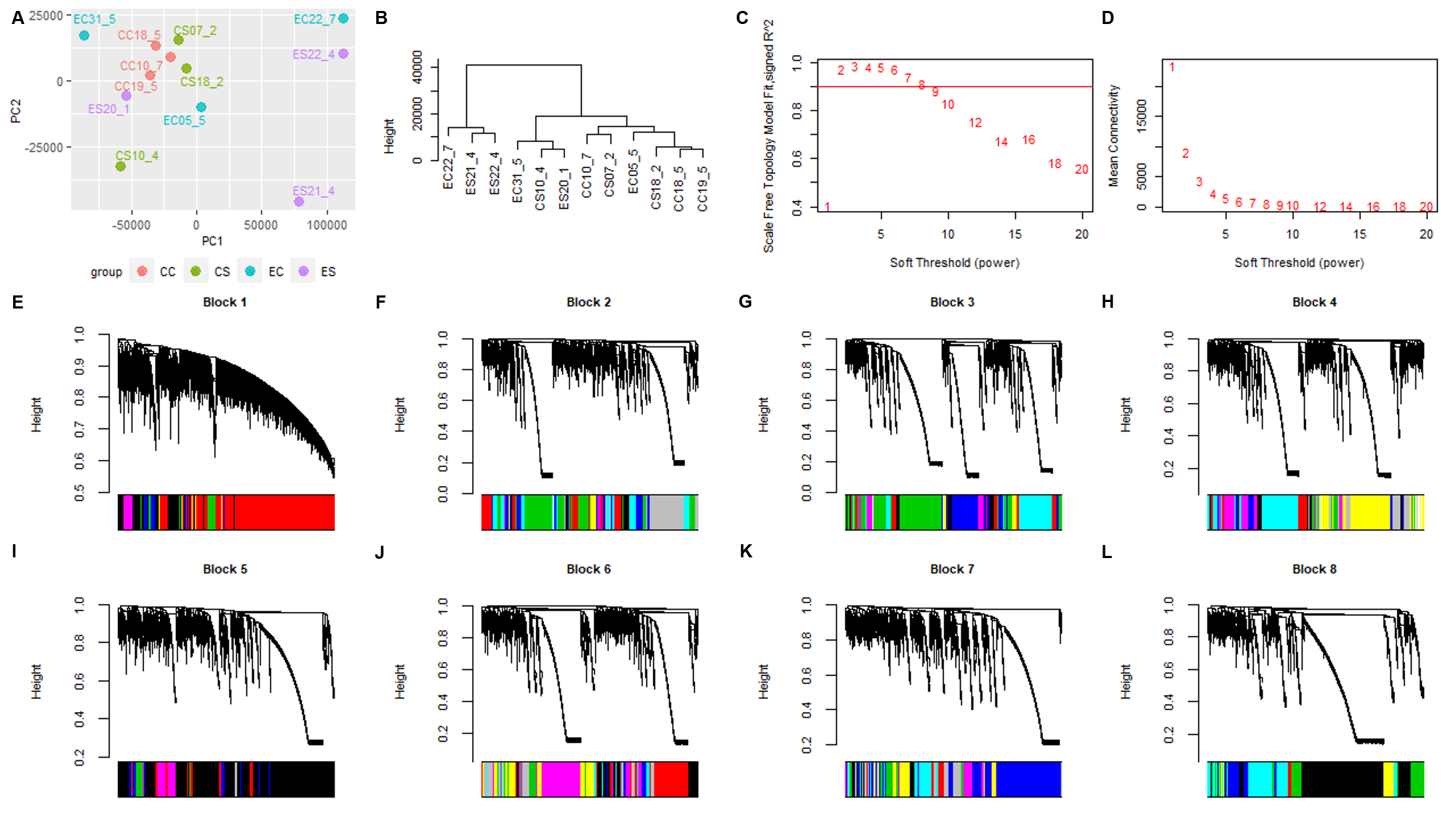

Supplement: Supplementary file 2 — Additional file 2: Supplementary Figure 1. Module creation by weighted gene co-expression network analysis. (A) Principal component analysis plot for principal components one and two. (B) Sample clustering to detect outliers. (C) Connectivity analysis of the scale-free topology fit for different soft-thresholding powers where numbers indicate the soft-thresholding power (D) mean connectivity of the network for different soft-thresholding powers, a soft-threshold of 9 was used here. (E, F, G, H, I, J, K, L) Transcript similarity clustering dendrograms for blockwise analysis for blocks 1-8, respectively, by clustering of transcripts based on topological overlap with different modules indicated by color below. [file 11689_2020_9316_MOESM2_ESM.tif]

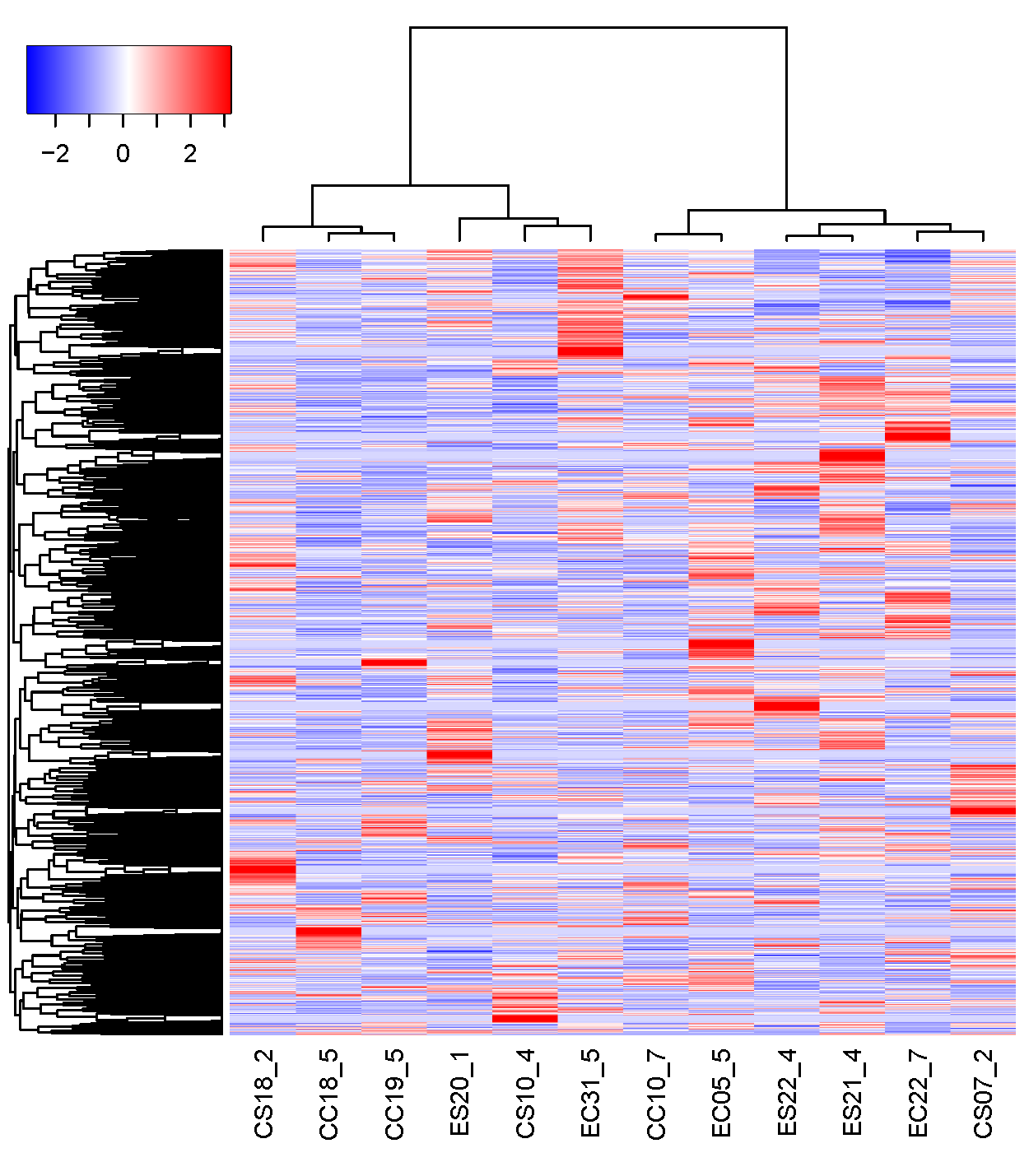

Supplement: Supplementary file 5 — Additional file 5: Supplementary Figure 2. Hierarchical clustering and heatmap of Module 19 gene transcript expression for each sample. [file 11689_2020_9316_MOESM5_ESM.tiff]

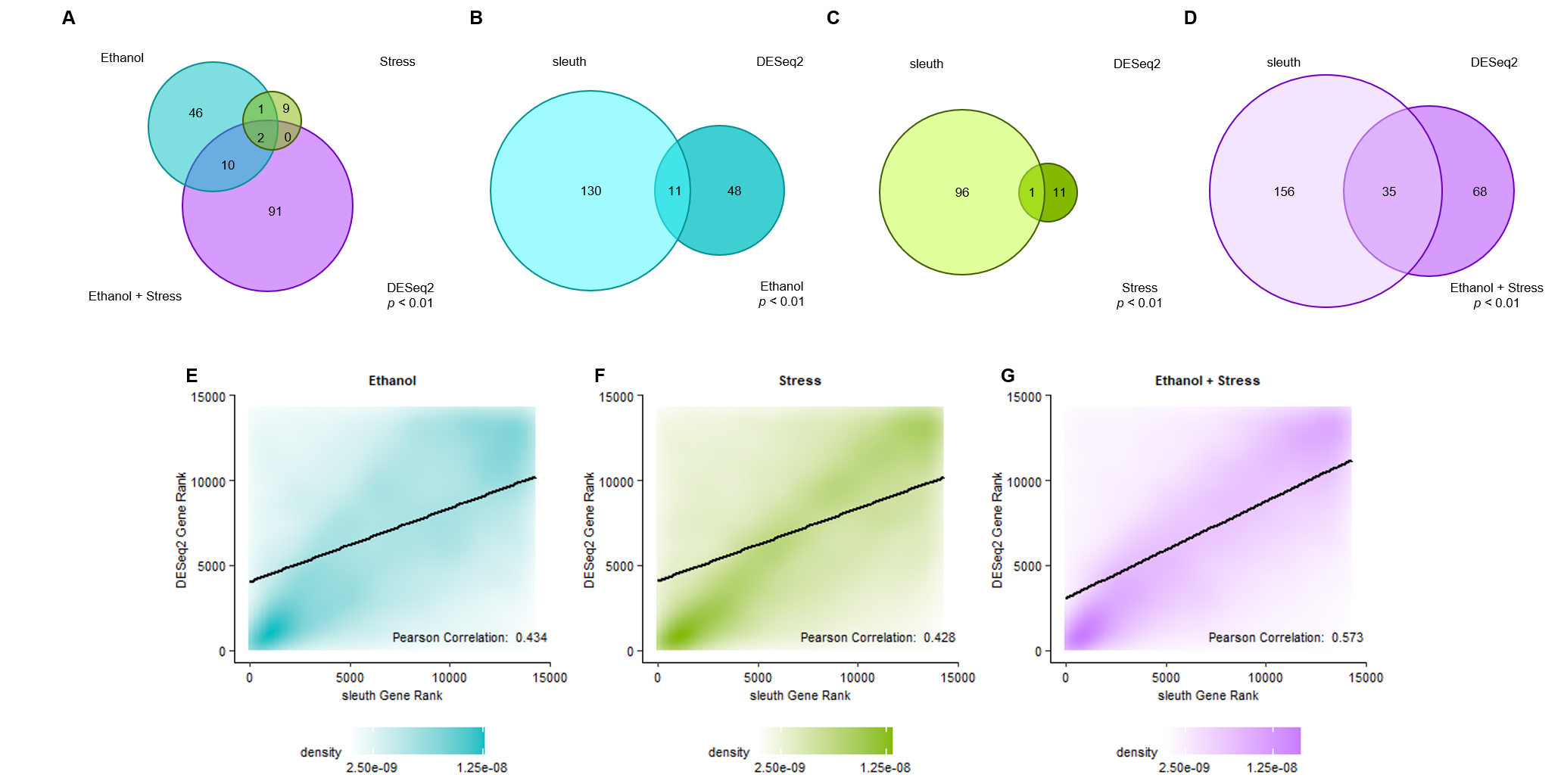

Supplement: Supplementary file 9 — Additional file 9: Supplementary Figure 3. Venn diagrams of overlapping differentially expressed genes (p < 0.01) for (A) each treatment group as detected by DESeq2, (B) Ethanol group as detected by sleuth and DESeq2, (C) Stress group as detected by sleuth and DESeq2, (D) Ethanol + Stress as detected by sleuth and DESeq2. Gene rank by p-value density plots between sleuth (x-axis) and DESeq2 (y-axis) analysis pipelines for (E) Ethanol (r = 0.434, p < 2.2 x 10^-16^), (F) Stress (r = 0.428, p < 2.2 x 10^-16^), and (G) Ethanol + Stress (r = 0.573, p < 2.2 x 10^-16^). [file 11689_2020_9316_MOESM9_ESM.tif]

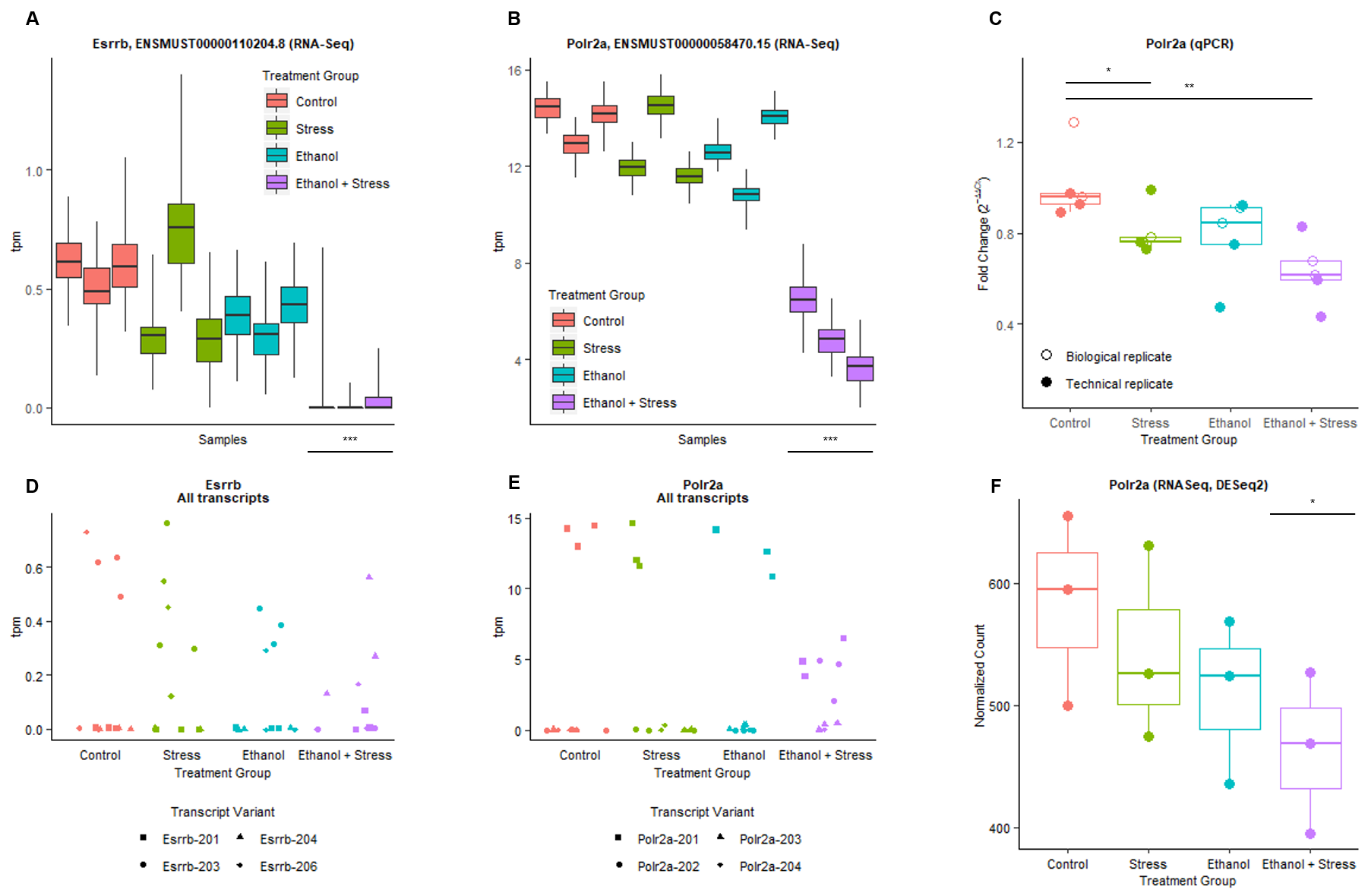

Supplement: Supplementary file 10 — Additional file 10: Supplementary Figure 4. Transcript abundance in transcripts per million (tpm) as detected by sleuth from RNA-Seq for (A) Esrrb-203 (ENSMUST00000110204.8) and (B) Polr2a-201 (ENSMUST00000058470.15). (C) The relative quantity of Polr2a is decreased 1.24 -fold with postnatal stress alone, and 1.59-fold when mice were prenatally exposed to ethanol and postnatal stress, as detected by reverse transcription qPCR, open circles represent biological replicates not used in the RNA-Seq experiment, while closed circles represent samples included in the RNA-Seq experiment and serve as technical replicates. Transcript abundance in transcripts per million (tpm) using the kallisto-sleuth pipeline for all detected transcript variants of (D) Esrrb and (E) Polr2a. Esrrb-203 (ENSMUST00000110204.8) and Polr2a-201 (ENSMUST00000058470.15) are down-regulated following Ethanol + Stress as compared to controls and represent the largest protein-coding transcripts for each gene. (F) Polr2a is decreased 0.80-fold when mice were prenatally exposed to ethanol and postnatal stress, presented as normalized counts determined using the HISAT2-featureCounts-DESeq2 pipeline, *p < 0.05, **p < 0.01, ***q < 0.01. Note: We have included panels A-C from Fig. 3 to facilitate comparison. [file 11689_2020_9316_MOESM10_ESM.tif]
